# Multiple myeloma cells recruit tumor-supportive macrophages through the CXCR4/CXCL12 axis and promote their polarization toward the M2 phenotype

**DOI:** 10.18632/oncotarget.2207

**Published:** 2014-07-12

**Authors:** Katia Beider, Hanna Bitner, Merav Leiba, Odit Gutwein, Maya Koren-Michowitz, Olga Ostrovsky, Michal Abraham, Hanna Wald, Eithan Galun, Amnon Peled, Arnon Nagler

**Affiliations:** ^1^ Hematology Division and CBB, Guy Weinshtock Multiple Myeloma Foundation, Chaim Sheba Medical Center, Tel-Hashomer, Israel; ^2^ Goldyne Savad Institute of Gene Therapy, Hadassah Hebrew University Hospital, Jerusalem, Israel; ^3^ Biokine Therapeutics Ltd., Science Park, Ness Ziona, Israel

**Keywords:** MM, M2 macrophages, CXCR4

## Abstract

Multiple myeloma (MM) cells specifically attract peripheral-blood monocytes, while interaction of MM with bone marrow stromal cells (BMSCs) significantly increased monocyte recruitment (p<0.01). The CXCL12 chemokine, produced by both the MM and BMSCs, was found to be a critical regulator of monocyte migration. CXCL12 production was up-regulated under MM-BMSCs co-culture conditions, whereas blockage with anti-CXCR4 antibodies significantly abrogated monocyte recruitment toward a MM-derived conditioned medium (p<0.01). Furthermore, elevated levels of CXCL12 were detected in MM, but not in normal BM samples, whereas malignant MM cells often represented the source of increased CXCL12 in the BM. Blood-derived macrophages effectively supported MM cells proliferation and protected them from chemotherapy-induced apoptosis. Importantly, MM cells affected macrophage polarization, elevating the expression of M2-related scavenger receptor CD206 in macrophages and blocking LPS-induced TNFα secretion (a hallmark of M1 response). Of note, MM-educated macrophages suppressed T-cell proliferation and IFNγ production in response to activation. Finally, increased numbers of CXCR4-expressing CD163+CD206+ macrophages were detected in the BM of MM patients (n=25) in comparison to MGUS (n=11) and normal specimens (n=8).

Taken together, these results identify macrophages as important players in MM tumorogenicity, and recognize the CXCR4/CXCL12 axis as a critical regulator of MM-stroma interactions and microenvironment formation.

## INTRODUCTION

Multiple myeloma (MM) is a B-cell neoplasm characterized by clonal expansion of malignant plasma cells in the BM compartment, where they proliferate and acquire resistance to chemotherapy-mediated apoptosis. MM accounts for 10% of malignant hematological diseases. With the introduction of novel agents, such as bortezomib and lenalidomide, the median survival was prolonged from 3-4 to 7 years. However, MM remains mostly incurable due to the development of drug resistance, which leads to relapsed/refractory disease [1, 2]. It was previously demonstrated that interaction of the malignant plasma cells with the BM microenvironment is critical for homing, survival and acquisition of MM cell drug-resistance [3, 4]. The BM milieu contains various components, including stromal cells (BMSCs), osteoclasts and immune cells. BMSCs were shown to promote growth and drug-resistance in MM cells [5]. However, the functional role of other components in the microenvironment is less clear. Reciprocal positive and negative interactions between plasma cells and the BM stroma are orchestrated by an array of cytokines, receptors and adhesion molecules [6, 7]. These observations suggest that both myeloma-derived and stromal cell-produced factors, such as chemokines, participate in the regulation of MM growth and progression [8]. Moreover, chemokines not only regulate the homing and re-circulation of MM cells, but also enhance tumor growth, vascularization and bone destruction [9-12].

Compelling evidence has emerged in recent years suggesting that macrophages play an important role in tumor development and progression. These highly heterogeneous myeloid cells can acquire a range of different phenotypes based on the environmental stimuli. In a simplified view, M1 (classically activated, inflammatory) and M2 (alternative, suppressive) types represent two extreme phenotypes on the polarization continuum [13]. Tumor-associated macrophages (TAMs) are the major components of tumor-infiltrating leukocytes that orchestrate various aspects of cancer, modulating the tumor environment by suppressing anti-tumor immune responses, inducing angiogenesis and promoting tumor progression. Generally, TAMs exhibit similarities with prototypic M2-polarized macrophages [14-16]. Understanding the key factors that modulate TAM infiltration and differentiation is important for elucidating the mechanisms underlying TAM-mediated tumor-promoting effects. In MM, macrophages have been reported to negatively impact disease course. It was previously reported that in patients with active MM, higher numbers of macrophages present in the BM environment and that macrophages contribute to neovasculogenesis [17, 18].

On the basis of these findings, myeloma-derived and stromal cell-produced factors, such as chemokines, could play an indispensable role in macrophage recruitment, differentiation, stroma-MM cell interactions, and disease progression. However, few studies have described the interactions of macrophages with MM cells.

Our recent findings implicate the important role of the CXCR4/CXCL12 chemokine axis in macrophage recruitment and cell-cell interactions with MM cells. In the present study, we propose to study the role of macrophages in MM, including their role in the tumor microenvironment formation and drug response.

## RESULTS

### Interaction between MM cells and BMSCs increases the recruitment of peripheral blood monocytes

**Figure 1:**
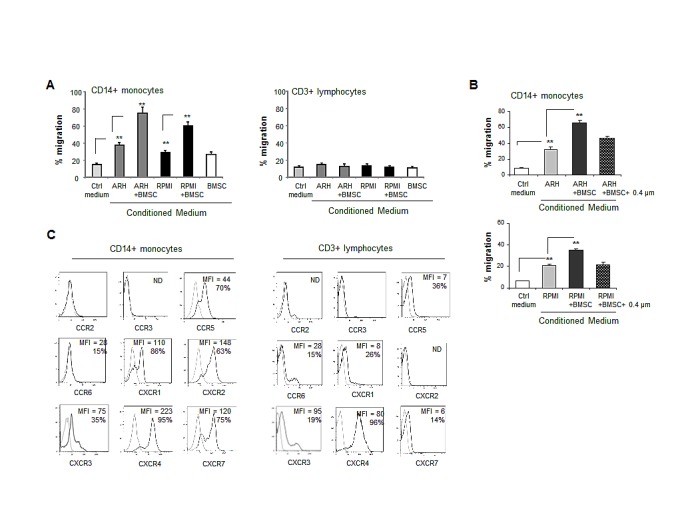
Interaction between MM and BMSCs induces the ability of MM cells to selectively attract peripheral blood monocytes (A) Trans-well migration of PBMCs toward conditioned medium (CM) produced by MM cells ARH77 and RPMI8226 cultured alone or in the presence of BMSCs. Migration of CD14-positive monocytes and CD3-positive lymphocytes was enumerated by FACS and percent of migrating cells out of total input cells was calculated. Data is presented as mean of triplicates ±STDEV (**p<0.01). Each experiment was repeated three times. (B) Migration of CD14+ monocytes in response to CM produced by MM cells incubated in either direct contact with BMSCs or separated with 0.4 μm membrane during the co-culture. Data is presented as mean of triplicates ±STDEV (**p<0.01). Each experiment was repeated three times. (C) Chemokine receptor expression repertoire on CD14+ monocytes or CD3+ lymphocytes evaluated by cell-surface co-staining of PBMCs and FACS analysis.

Trafficking of immune cells to the BM of MM patients and their localization in the tumor site are one of the first critical steps in a cascade of events that may shape the MM microenvironment and may affect disease development and progression. We, therefore, explored the in vitro migration of PBMCs in response to conditioned medium (CM) produced by ARH77 and RPMI8226 MM cell lines, cultured alone or co-cultured with BMSCs. Analysis of the migrated immune cell populations (CD3+ T cells, CD19+ B cells and CD14+ monocytes) was performed in order to identify the cells responding to the chemotactic stimuli provided by MM cells. As demonstrated in Figure 1A, MM-produced CM specifically induced enrichment in the monocyte fraction migrating toward the CM produced by MM cells in vitro. Co-culture of MM cells with BMSCs resulted in a further significant increase in monocyte recruitment toward CM (Fig. 1A). Interestingly, separation between MM cells and BMSCs with transwells abrogated the contribution of BMSCs to enhanced monocyte migration, since the migration capability was similar to that achieved by the CM produced by MM cells alone (Fig. 1B). These results suggest that the contact-dependent interaction between MM and BMSCs is required for chemokine induction expression and the subsequent increased monocyte migration.

Next, the repertoire of chemokine receptors expressed on the surface of migrating monocytes and T cells was analyzed. Differential expression of chemokine receptors was observed. Out of nine receptors tested, CD3+ T cells mainly expressed CXCR4 (96% of cells, MFI 80), as well as low levels of CCR5, CXCR1 and CXCR7. A small proportion of cells (15%) expressed CCR6. In contrast, CD14+ monocytes highly expressed CCR5 (70% of cells), CXCR1 (86%) and CXCR2 (63%). Furthermore, higher levels of CXCR4 (95% of cells, MFI 226) and CXCR7 (75% of cells, MFI 120) were detected in monocytes compared to T lymphocytes (Fig. 1C). These results suggest the potential ability of monocytes to respond to a wide range of chemotactic stimuli, including the CCR5, CXCR1/CXCR2 and CXCR4/CXCR7 axes.

### MM cells represent a significant source of CXCL12 in the MM BM niche, while interaction with BMSCs stimulates the CXCL12 production by MM cells

**Figure 2:**
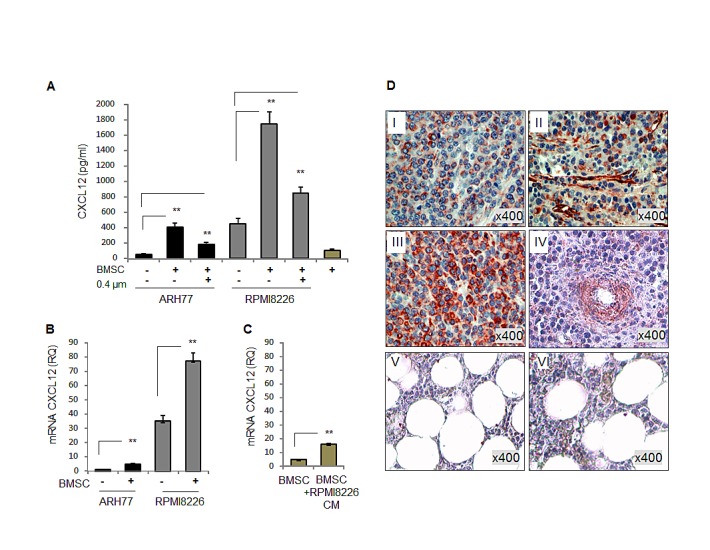
Both MM and BMSC cells express CXCL12. Interaction with BMSCs elevates CXCL12 expression in MM cell lines (A) Secretion of CXCL12 by MM cell lines RPMI8226 and ARH77, incubated in the absence or presence of BMSCs, in direct contact or separated by 0.4 μm-transwells for 48 hours, evaluated by ELISA. (B) Expression of CXCL12 mRNA in MM cells RPMI8226 and ARH77, incubated in the absence or presence of BMSCs for 48 hours, evaluated by quantitative PCR. (C) Expression of CXCL12 mRNA in human BMSCs, untreated or treated with RPMI8226-produced CM, evaluated by quantitative PCR. Data is presented as mean of triplicates ±STDEV (**p<0.01). (D) Expression of CXCL12 in four MM (I, II, III and IV) and two normal (V and VI) BM samples evaluated by immunohistochemical staining. Original magnification of x400 is shown.

The presence of high levels of CXCR4 and CXCR7 receptors in monocytes led us to study the possible role of the corresponding chemokine ligand, CXCL12, in myeloma-induced monocyte recruitment. CXCL12 is an abundant component that is present in the BM milieu, and is constitutively expressed by BMSCs [19]. In agreement with a previous report [20], here we demonstrate that in addition to BMSCs, MM cells can be a significant source of CXCL12 in the BM environment, therefore co-expressing both the receptor CXCR4 and the ligand CXCL12. CXCL12-producing MM cell lines, RPMI8226 and ARH77, were co-cultured with human BMSCs, in direct contact or separated by 0.4 μm transwells, and CXCL12 levels in the medium were measured. A significant increase in CXCL12 secretion was detected in the co-cultures allowing direct contact between MM cells (p<0.001), while in co-cultures with the transwell insert, lower levels of CXCL12 were observed (Fig. 2A). These results indicate that both contact-dependent and soluble factors are involved in the stroma-induced CXCL12 secretion. To examine whether BMSCs stimulate the production of CXCL12 by MM cells, mRNA levels of CXCL12 were tested in ARH77 and RPMI8226 cultured in the absence or presence of BMSCs. Elevated levels of the CXCL12 mRNA were observed in both MM cell lines upon co-culture with BMSCs (Fig. 2B). Importantly, only modest increase in CXCL12 transcription was detected in BMSCs treated with culture supernatants of RPMI8226 cells (Fig. 2C).

Next, the CXCL12 expression was evaluated in primary BM samples from MM patients and healthy controls. A commercially available tissue array of BM samples from MM patients was subjected to immuno-histochemical staining of CXCL12. As depicted in figure 2D, high levels of CXCL12 expression were detected in both MM cells and stromal cells from MM patients. In contrast, normal BM samples demonstrated lower levels of CXCL12.

These data indicate that the interaction between MM and BMSCs results in a reciprocal mutual activation that prompts an elevated CXCL12 production from both cell sources.

### Monocytes utilize the CXCR4/CXCL12 axis in their migratory response to MM- and BMSCs-produced signals

**Figure 3:**
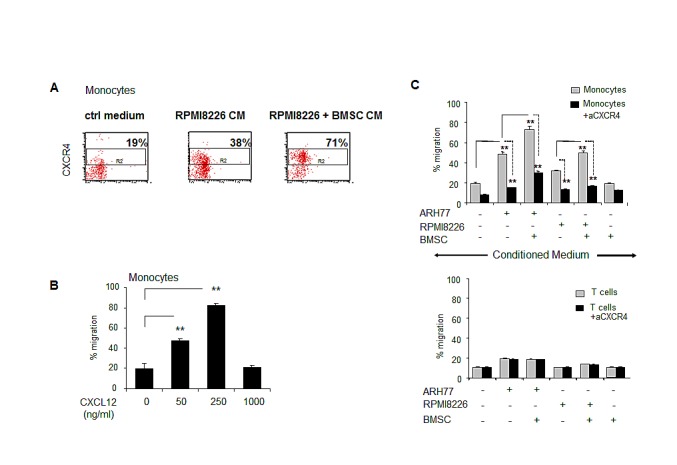
Peripheral blood monocytes respond in a CXCR4-dependetnt manner to MM-induced migratory signals and demonstrate dose-dependent migratory ability in response to CXCL12 PBMCs were allowed to migrate for 4 hours in response to CM produced by MM cell lines (ARH77 and RPMI8226), incubated in 1% FCS-containing medium in the absence or presence of BMSCs. (A) Quantification of migrated CD14+ CXCR4+ monocytes toward control medium or RPMI8226-produced CM was evaluated by FACS. Percent of CXCR4-positive cells out of total migrating CD14+ monocytes is presented. (B) PBMCs were allowed to migrate for 4 hours in response to elevated doses of recombinant human CXCL12. Quantification of migrated CD14+ monocytes was performed by FACS. Percent of migrated cells was calculated. Data is presented as mean of triplicates ±STDEV (**p<0.01). (C) To inhibit CXCR4, neutralizing anti-CXCR4 monoclonal antibody (20 μg/ml) was added to PBMCs in the upper chamber. Quantification of migrated CD14+ monocytes or CD3+ lymphocytes was performed using specific staining and FACS evaluation. Percent of migrated cells was calculated. Data is presented as mean of triplicates ±STDEV (**p<0.01). Each experiment was repeated twice.

To delineate the ability of CXCL12 produced by the MM cells to attract peripheral-blood monocytes, we first evaluated the fraction of CXCR4-expressing CD14-positive monocytes that responded to MM-produced CM. PBMCs were allowed to migrate in response to control medium or CM produced by RPMI8226 cultured in the absence or presence of BMSCs and migrating cells were collected and analyzed. CD14+ cell population was gated in and percent of CXCR4-expressing cells was determined. As demonstrated in figure 3A, enrichment in percent of CXCR4+ monocytes in the cell fraction migrating toward RPMI8226-produced CM was clearly observed (31% versus 19% in control). Furthermore, CM produced by RPMI8226 cells co-cultured with BMSCs resulted in additional attraction of CXCR4-expressing monocytes, reaching 71% of all migrating monocytes (Fig. 3A). Moreover, recombinant CXCL12 also potently induced the dose-dependent migration of monocytes (Fig. 3B). Next, we evaluated the effect of the CXCR4 blockade on the migratory potential of PBMCs. Pre-treatment with neutralizing anti-CXCR4 antibody significantly abrogated CD14+ monocyte recruitment in response to the CM produced by MM cells cultured either alone or in the presence of BMSCs. In contrast, CD3+ T cell migration was not affected by CXCR4 inhibition (Fig. 3C). Altogether, these findings suggest that CXCR4 may be critically involved in the MM-induced recruitment of monocytes.

### Macrophages effectively support the proliferation and survival of MM cell lines and primary MM cells, protect MM from drug-induced cell death and activate the expression of pro-inflammatory and pro-angiogenic factors in MM cells

**Figure 4:**
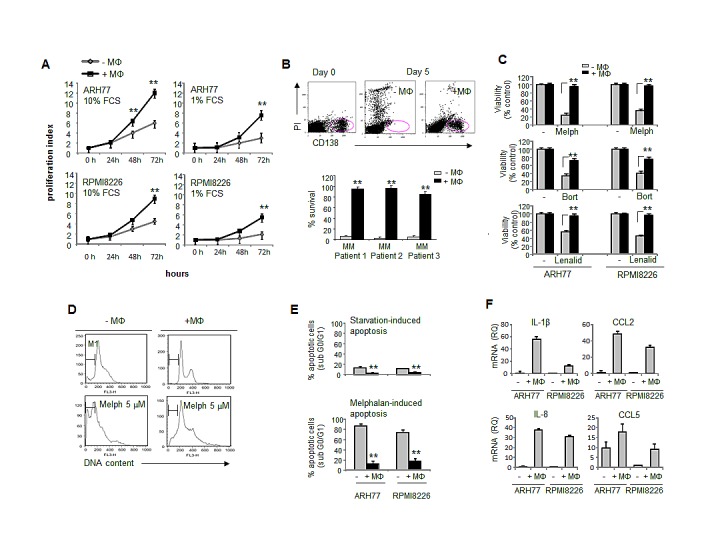
Macrophages support MM cell survival and proliferation, protect MM cells from chemotherapy-induced apoptosis and elevate expression of pro-angiogenic factors in MM cells (A) CFSE-labeled MM cells ARH77 and RPMI8226 were co-cul. tured under serum-reduced (1% FCS) or full-serum (10%) conditions in the absence or presence of peripheral blood-derived macrophages for 24, 48 and 72 hours. Viable MM cell number was determined by FACS using PI exclusion. Proliferation index was calculated. Data is presented as mean of triplicates ±STDEV (**p<0.01). (B) BM samples (n=3) from MM patients containing CD138+ cells were cultured in 10% FCS medium in the absence or presence of macrophages for 5 days and percent of viable CD138+ PI-negative plasma cells was detected by FACS. Data is presented as mean of triplicates ±STDEV (**p<0.01).(C) Viability of MM cells ARH77 and RPMI8226, treated with melphalan (5 μM), bortezomib (5nM) or lenalidomide (10 μM) in the absence or presence of peripheral blood-derived macrophages for 48 hours, evaluated by the XTT method. Data is presented as mean of triplicates ±STDEV (**p<0.01). (D, E) RPMI8226 cells were cultured under serum-reduced (1% FCS) conditions, with or without melphalan (5 μM), in the absence or presence of peripheral blood-derived macrophages. Cell cycle distribution was evaluated and percent of apoptotic (sub G0/G1) cells was detected. (D) Representative images demonstrating cell cycle distribution. (E) Quantification of apoptotic DNA fragmentation (percent of sub G0/G1 population). Data is presented as mean of triplicates ±STDEV (**p<0.01).(F) Expression of IL-1β, IL-8, CCL2 and CCL5 mRNA in MM cells RPMI8226 and ARH77, incubated in the absence or presence of peripheral blood-derived macrophages for 48 hours, evaluated by quantitative PCR.

Following migration and tissue recruitment, monocytes undergo differentiation and become macrophages. Therefore, the functional consequence of myeloma-macrophage interactions on the behavior of myeloma cells was assessed. Blood-derived macrophages significantly increased the proliferation of MM cell lines under both normal and serum-reduced conditions (Fig. 4A). Moreover, macrophages supported the in vitro survival of primary CD138+ cells isolated from the BM of MM patients. As demonstrated in figure 4B, 80-90% of primary CD138+ cells underwent apoptosis following two days of culture, whereas the presence of macrophages protected the CD138+ cells from apoptosis resulting in a 90% cell survival.

The protective role of BMSCs is a well characterized phenomenon in MM. However, the role of macrophages in the protection of MM cells from chemotherapy- and immune modulating drug-induced apoptosis is less clear. We therefore evaluated the putative role of macrophages in MM cell response to anti-myeloma agents including melphalan, bortezomib and lenalidomide. Importantly, incubation with macrophages significantly protected the MM cells from drug-induced cell death (Fig. 4C), indicating a possible role of macrophages in the MM response to chemo- and immunotherapy. Incubation with macrophages protected MM cells from both starvation- and chemotherapy-induced apoptosis and significantly reduced apoptotic DNA fragmentation (Fig. 4D, E).

The interaction between cancer and accessory cells can activate the release of tumor-promoting cytokines and growth factors, establishing a tumor-favorable microenvironment. Therefore, we tested whether macrophages affect the expression of different pro-inflammatory cytokines in MM cells. Indeed, we were able to show that macrophages strongly induced at the mRNA level the expression of the chemokines CCL2 and CCL5, the pro-inflammatory cytokine IL-1β and the pro-angiogenic factor IL-8 in MM cells (Fig. 4F).

Altogether, these data demonstrate the critical role of macrophages in myeloma growth, survival, expression of inflammatory and angiogenic factors and drug resistance.

### MM cells affect the macrophage phenotype and promote M2 polarization

**Figure 5:**
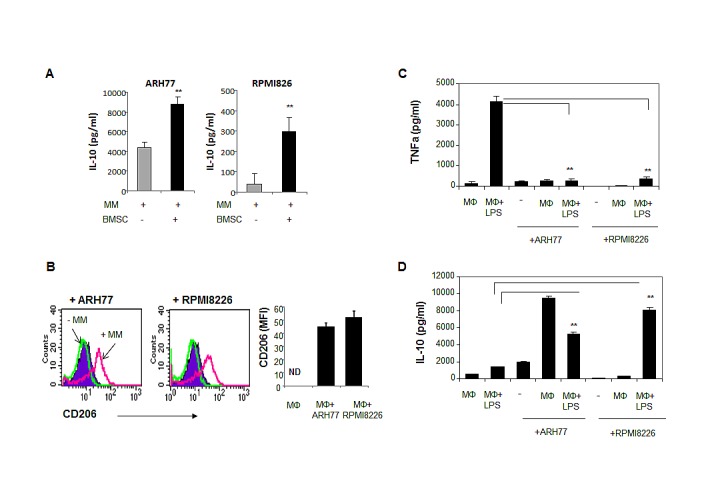
MM cells affect macrophage polarization, promote M2 phenotype acquisition and block M1 response in LPS-treated macrophages (A) MM cells ARH77 and RPMI8226 were incubated in the absence or presence of BMSCs for 48 hours and IL-10 secretion to culture medium was measured using ELISA kit. Data is presented as mean of triplicates ±STDEV (**p<0.01). (B) Peripheral-blood generated macrophages were incubated in the absence or presence of MM cells ARH77 and RPMI8226 for 48 hours, and surface expression of CD206 on CD14-expressing macrophage cell population was evaluated using flow cytometry analysis. (C, D) Effect of MM education on M1 stimuli response (LPS treatment) in macrophages. Peripheral-blood generated macrophages were pre- incubated in the absence or presence of MM cells ARH77 and RPMI8226 for 24 hours; non-adherent MM cells were removed and cells were stimulated with LPS (100 ng/ml) for an additional 48 hours. Medium was collected and levels of TNFα (C) and IL-10 (D) were measured using ELISA commercial kits.

The tumor-promoting activities of macrophages are related to their alternative activation (M2 phenotype) [21, 22]. Macrophage polarization is affected by the tumor microenvironment. IL-10 secretion by tumor cells was previously demonstrated as a key factor contributing to M2 polarization [15, 21]. Accordingly, we observed that MM cells produce significant amounts of IL-10, with higher levels obtained following interaction with BMSCs (Fig. 5A). To assess the ability of MM cells to educate macrophages and affect their polarization, we evaluated the expression of CD206, one the M2 hallmarks, on the cell surface of macrophages cultured in the absence or presence of MM cells. Co-culture with both ARH77 and RPMI8226 significantly up-regulated CD206 expression on macrophages (Fig. 5B).

Next, we wondered whether MM cells can affect macrophage polarization in response to M1 stimuli. Macrophages were cultured in the absence or presence of MM cells and then challenged with LPS, the canonical M1 stimulator. In macrophages cultured without pre-incubation with MM cells, LPS activation resulted in a predictable M1 response, as demonstrated by a significant increase in TNFα secretion. However, pre-incubation of macrophages with ARH77 or RPMI8226 cells completely abrogated TNFα secretion in response to LPS stimulation (Fig. 5C). In contrast, the production of IL-10, the cytokine characterizing the M2 activation profile, was significantly increased upon co-culture with MM cells and subsequent LPS stimulation (Fig. 5D). These results indicate that MM cells are able to educate macrophages, shifting their phenotype toward a M2-like activation profile.

### MM-educated macrophages suppress autologous T cell proliferation

**Figure 6:**
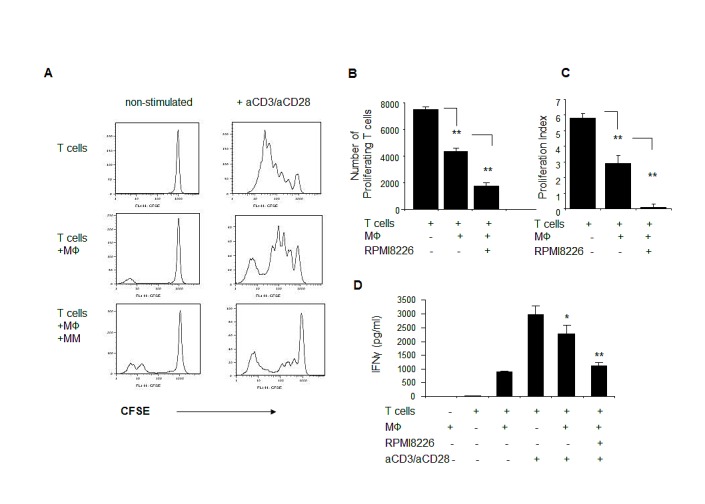
MM-educated macrophages suppress proliferation and IFNγ secretion of autologous T cells in response to polyclonal stimulation Peripheral-blood derived macrophages were pre-cultured in the absence or presence of RPMI8226 cells for 48 hours, and excess of myeloma was removed by pipetting. Frozen lymphocyte-enriched autologous PBMCs were thawed, labeled with CFSE (5 μM), plated in the absence or presence of macrophages and stimulated with anti-CD3 (OKT3) (10 μg/ml) and anti-CD28 (1 μg/ml) antibodies for 5 days. Cell division was monitored by FACS. Number of cell divisions and relative number of proliferated cells was measured based on the reduction in CFSE intensity. (A) Representative histogram plots showing decrease in the proliferation rate of stimulated T cells in the presence of MM-educated macrophages. (B) Numbers of proliferating T cells were quantified. Data is presented as mean of triplicates ±STDEV (**p<0.01). (C) Proliferation index of polyclonally-stimulated T cells, calculated as described in methods section. (D) IFNγ secretion to the culture medium, collected at the end of proliferation (day 5) and measured using ELISA. Data is presented as mean of triplicates ±STDEV (**p<0.01).

To further evaluate the potential of myeloma-educated macrophages to shape an immune response, the effect of macrophages on T cell proliferation was tested. CD3+ T cells were polyclonally activated with anti-CD3 and anti-CD28 antibodies in the absence or presence of autologous naïve or MM-primed macrophages. The T cell proliferative response was evaluated using the CFSE tracking method. Suppression of T cell proliferation was observed when T cells were co-cultured with autologous macrophages. Moreover, the addition of MM cells to macrophages two days prior to T cell proliferation further decreased T cell proliferation (p<0.001) (Fig. 6A-C).

One of the markers of T cell proliferation and activation is IFNγ secretion. Therefore, we evaluated the effect of macrophages on T cell IFNγ production in response to anti-CD3/anti-CD28 stimulation. As depicted in figure 6D, macrophages markedly reduced the levels of secreted IFNγ upon polyclonal activation. Furthermore, MM-primed macrophages demonstrated an enhanced suppressive potential (p<0.01).

Together, our findings indicate an immunosuppressive role for MM-educated macrophages resulting in skewed T cell function, as demonstrated by a decreased proliferation and a reduced IFNγ production upon polyclonal activation.

### M2 macrophages are significantly elevated in the BM of MM patients

**Figure 7:**
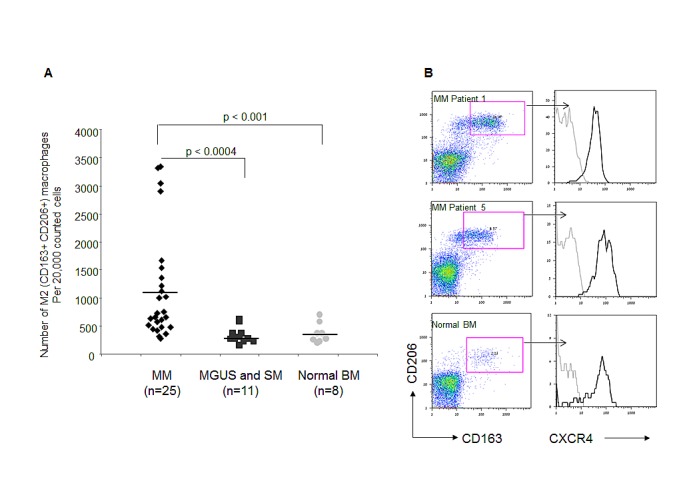
Elevated numbers of CXCR4-expressing M2 macrophages in BM samples of MM patients The presence of CD163+ CD206+ macrophages was assessed in BM samples from patients with MM, smoldering myeloma, MGUS and normal controls using FACS analysis. (A) Numbers of CD163+ CD206 + macrophages assessed in 25 patients with MM, 11 patients with MGUS and smoldering MM, and 8 normal controls (out of 20,000 cells acquired by FACS). (B) Representative plots demonstrating the M2 population on two MM samples and one normal control. Histograms represent CXCR4 surface levels expressed by CD163+ CD206+ macrophages.

To evaluate the potential clinical relevance of our findings, the presence of M2 macrophages (CD163+ CD206+) was determined in BM samples from MM patients, and compared with samples from patients with smoldering myeloma (SM), monoclonal gammopathy of undetermined significance (MGUS) and healthy volunteers. Patient characteristics are presented in Table 1. The number of CD163+CD206+ M2 macrophages was significantly elevated in the BM of patients with MM when compared with the BM samples from patients with SM and MGUS (p<0.0004), as well as with normal controls (p<0.001) (Fig. 7A). Importantly, this macrophage population expressed reliable levels of cell-surface CXCR4, as shown in figure 7B by the representative staining from two MM BM samples and one normal BM. The level of CXCR4 expressed by M2 macrophages from MM BM samples was similar to that expressed by M2 macrophages present in a normal BM sample. Taken together, these findings indicate the presence of increased numbers of CXCR4-expressing M2 macrophages in the BM of MM patients, and suggest CXCR4 as one of the possible routes of their recruitment.

**Table 1:** Patient characteristics

Characteristic	MM (n=25)	MGUS and Smoldering MM (n=11)	Mann-Whitney U test
Median age, years (range)	58.2 (39 - 77)	60.5 (57 - 69)	
Gender Male Female	16 (64%) 7 (36%)	6 (54.5%) 5 (45.5%)	
Myeloma type IgG IgA Light chain only	14 8 7	5 4 2	
Hb, g/dL mean (range)	11.4 (8-14.6)	13.7 (12.9-15)	p<0.001
B2M, mg/L mean (range)	4.89 (1.8-11.4)	3.7 (2-5.5)	p<0.01
Serum M-protein, g/dL mean (range)	3.28 (1.7-12)	1.2 (0.2-2.5)	p<0.001

Abbreviations: Hb – hemoglobin, B2M – beta-2-microglobulin.

## DISCUSSION

Macrophages are key components of the myeloid infiltrate in tumors [23]. Increasing evidence suggests TAMs as important players in tumor progression and TAM infiltration could emerge as a potentially useful prognostic marker [24]. The mechanism of tumor promotion by TAMs was mostly established in solid tumors. Myelomonocytic cells influence almost all steps of carcinogenesis, contributing to genetic alterations and instability, promoting angiogenesis, suppressing adaptive immunity, remodeling extracellular matrix and promoting invasion and metastasis [25].

Our study shows that MM cells are capable of secreting chemo-attractive factors that specifically recruit blood-derived monocytes. Interaction of MM cells with BMSCs further increases monocyte recruitment in vitro. Analysis of chemokine receptors expressed by responder monocytes demonstrated high levels of surface CXCR4 and CXCR7, revealing them as possible candidates that may regulate MM-induced monocyte recruitment. Indeed, CXCR4 inhibition with neutralizing antibodies significantly suppressed monocyte migration in response to MM-produced factors.

Accumulating evidence implicates the important role of CXCL12 in monocyte recruitment and function. It was previously shown that both CXCR4 and CXCR7 are necessary for CXCL12-mediated migration of monocytes, and that CXCL12 promotes monocyte differentiation toward a pro-angiogenic and immunosuppressive phenotype [26].

Tumor-derived CXCL12 was shown to attract tumor-promoting myeloid CD11b+ cells in a mouse model of Lewis lung carcinoma [27]. Furthermore, CXCL12 expressed by colon cancer metastasis to the liver was demonstrated to promote crosstalk between cancer cells and macrophages, inducing tumor-favorable GM-CSF/HB-EGF paracrine loop [28]. As for MM, Zannettino and colleagues reported that MM cells produced CXCL12, thus mediating bone resorption and promoting osteolytic bone disease [20]. Our study recognizes MM cells (in addition to BMSCs) as a considerable source of CXCL12 in the tumor niche. The presence of BMSCs, however, significantly contributes to the elevated CXCL12 levels. In addition to their endogenous CXCL12 production, BMSCs enhanced the production of CXCL12 by MM cells, as demonstrated by both ELISA and quantitative PCR. It is conceivable that CXCR4-dependent macrophage recruitment to the MM niche may further augment the vicious cycle of CXCL12 secretion. Notably, it was recently shown that BM macrophages are able to augment CXCL12 production by BMSCs, therefore contributing to progenitor retention [29].

The ability of CXCL12 to recruit suppressive myeloid cells was demonstrated in a glioblastoma tumor model. Pharmacological inhibition of HIF1α or the CXCL12/CXCR4 axis reduced BM-derived cell recruitment and prevented tumor recurrence [30, 31]. These findings suggest that the hypoxic environment characterizing the BM niche may further support CXCL12 expression and the subsequent accumulation and retention of suppressor myeloid cells.

There is evidence supporting the hypothesis that myeloma-associated macrophages are recruited from peripheral blood rather than develop from BM-resident monocytic precursors. Extramedullar plasmacytomas are rich in TAMs [32]. Another recent work demonstrated that due to high levels of cytostatic cytokines, such as TGFβ, myelopoiesis is suppressed in the BM environment of MM patients [33].

Macrophages display plasticity in both phenotype and function. Macrophage polarization is dependent on environmental signals. It is thus conceivable that once recruited to the MM microenvironment, monocytes are exposed to tumor- and stroma-secreted factors (such as IL-10, hypoxia) and may acquire characteristics of alternative M2 polarization. M2 macrophages are suited to promote tumor progression, supporting tumor cell proliferation, enhancing angiogenesis and shaping the immunosuppressive microenvironment.

Our results show that blood-derived macrophages support MM cell survival, proliferation and protect MM from drug-induced apoptosis. Furthermore, interaction with macrophages induces a pro-angiogenic and pro-inflammatory factors expression in MM cells. Reciprocally, MM cells were shown to affect macrophage polarization, inducing the expression of the M2 marker CD206. The presence of MM cells suppresses M1-associated TNFα secretion and promotes M2-related IL-10 production in response to TLR4 stimulation with LPS. These findings are in accordance with studies showing that phagocytosis of apoptotic tumor cells by macrophages inhibited LPS-induced TNFα and IL-6 secretion, but not IL-10 secretion [34]. Endogenous ligands for TLR4 such as hyalouronic acid (HA) and heat shock protein 60 (HSP60) are abundantly present in malignant tumors [35, 36]. Therefore, chronic activation of TLR4 on TAMs in the tumor niche can induce M2 rather than the M1 phenotype.

In addition to the direct effect of macrophages on MM proliferation and drug resistance, our results indicate that interaction between macrophages and MM may shape the immune responses towards immune suppression. We observed that MM cells enhance the ability of macrophages to suppress the proliferation and activation of polyclonally-stimulated autologous T cells. Immune dysfunction is a well-documented phenomenon in MM patients [37]. Recent studies report that MM cells can suppress immunity by various mechanisms, including up-regulation of co-inhibitory molecules such as PD1-L and CD200 [38]. Another way to evade the immune system is the generation of the immunosuppressive microenvironment by secretion of immune inhibitory factors like IL-10 or TGFβ that impair effector cell activation and recruit regulatory T cells (T reg) [39]. T reg accumulation was also detected in MM patients following allogeneic stem cell transplantation [40]. The immunosuppresory role of macrophages in MM is less established. Here we demonstrate a direct immuno-inhibitory effect of MM-educated macrophages, implicating their role in the suppression of normal T cell responses in MM.

Accumulation of suppressive macrophages in the MM tumor site may negatively affect the response to anti-MM therapy and clinical outcome. Recently, it was shown that increased numbers of M2 macrophages, characterized by expression of CD163 and CD68 in the bone marrow of 68 MM patients, were associated with an unfavorable outcome [41].

Taken together, our findings indicate that macrophages are important players in MM growth and chemo-resistance. Furthermore, this study demonstrates that macrophages are recruited and polarized by signals emitted by MM and BMSCs and recognize the CXCR4/CXCL12 axis as a critical regulator of MM-stroma interactions, shaping the MM tumor niche and contributing to the microenvironment formation.

## MATERIALS AND METHODS

### Cell lines and MM patient samples

Human MM cell lines were obtained from American Type Culture Collection (Rockille, MD, USA): ARH77, RPMI8226. Cells were maintained in log-phase growth in RPMI 1640 medium (Biological Industries) supplemented with 10% heat-inactivated fetal calf serum (FCS), 1mM L-glutamine, 100 U/ml penicillin, and 0.01 mg/ml streptomycin (Biological Industries) in a humidified atmosphere of 5% CO_2_ at 37^0^C.

Primary MM cells were isolated from bone marrow aspirates of consenting myeloma patients. Mononuclear cells were collected after standard separation on Ficoll-Paque (Pharmacia Biotech).

### Preparation of BMSCs and co-culture experiments

Primary human bone marrow stromal cells (BMSCs) were produced from bone marrow aspirates of healthy donor volunteers after signing an informed consent. BMSCs were isolated by plate adherence and expanded as previously described [42]. For co-culture, MM cells were either seeded on top of the stromal cells or separated with 0.4 μm-pore transwells (Costar). Following 48 hours, cells and conditioned medium were collected for subsequent analysis.

### Preparation of macrophages

Peripheral blood mononuclear cells (PBMCs) were obtained from the blood of consenting healthy donor volunteers by Ficoll-Paque density centrifugation and allowed to adhere to culture plates for 2 hours at 37^0^ C. Non-adherent lymphocyte-enriched cells were removed and cryopreserved for T cell proliferation assay. The adherent monocytes were incubated for 10 days in DMEM medium supplemented with 10% heat-inactivated FCS, 100 U/ml penicillin, 100 μg/ml streptomycin, 2mM L-glutamine and sodium pyruvate. Medium was changed and non-adherent cells were discarded every two days. Purity of monocyte-derived cells was verified by flow cytometry using the CD14 marker expression analysis and was > 95%.

### Flow cytometry analysis

The expression of CD3, CD14, CCR2, CCR3, CCR5, CCR6, CXCR1, CXCR2, CXCR3, CXCR4 and CXCR7 on the surface of PBMCs was evaluated using specific monoclonal antibodies. Expression of CD206 on macrophages cultured alone or in the presence of RPMI8226 MM cells was evaluated by co-staining with CD11b. Frequency of M2 macrophages in primary MM samples was evaluated by co-staining for CD163 and CD206 markers. All antibodies including appropriate isotype control were purchased from eBioscience. The cells were analyzed by FACScalibur (Becton Dickinson Immunocytometry Systems), using the CellQuest and FlowJo software.

### Immunohistochemistry

Commercial MM BM tissue microarray containing four cases of myeloma and two normal BM tissue samples was used (US Biomax). Formalin-fixed, paraffin-embedded tissue samples were initially dewaxed, rehydrated, treated with EDTA buffer and blocked with CAS blocking reagent (Zymed Laboratories) for 30 minutes atroom temperature. Samples were then incubated overnight at 4°C in a humidified chamber with anti-human CXCL12 antibody (R&D Systems), and diluted to a final concentration of 10 μg/ml. Next, the sections were incubated with secondary anti-mouse horseradish peroxidase-conjugated antibody (DakoCytomation) for 30 minutes at room temperature. 3-amino-9-ethylcarbazole (AEC) was used for color development, and sections were counterstained with hematoxylin.

### Cell migration assay

Migration assay was performed in triplicates using 5-μm pore size Transwells (Costar). The lower compartment was filled with 600 μl of conditioned medium produced by MM cells cultured in the absence or presence of BMSCs, or 1% FCS RPMI 1640 medium containing CXCL12 (50-500 ng/ml) (PeproTech EC), and 5×10^5^PBMCs in 100 μl of 1% FCS RPMI1640 medium were applied to the upper compartment. The numberof cells migrating within 4 hours to the lower compartment was determined by FACS using CD3 and CD14 differential staining and expressed as a percentage of the input. For CXCR4 neutralization, PBMCs were pre-treated with anti-CXCR4 antibody (10 μg/ml) (R&D Systems) for 30 minutes prior to migration.

### RT-PCR analysis

Total RNA from cells was extracted using Trizol reagent (Invitrogen) according to the manufacturer's instructions. Subsequently, cDNA was generated from 1 μg of total RNA using High-Capacity cDNA Reverse Transcription Kit (Applied Biosystems). mRNA levels were evaluated using SYBR Green quantitative PCR. The qPCR reaction contained 100 ng of total RNA-derived cDNAs, forward and reverse primers (300 nM) and PerfeCta SYBR Green FastMix (Quanta Biosciences), and was performed using the StepOnePlus Real Time PCR system (Applied Biosystems). Changes in expression levels were normalized to control β2-microglobulin using the ΔΔ*C _T_* method of relative quantification using the StepOne Software v2.2. Experiments were performed in triplicates for each sample. The sequences of primers are presented in Supplementary Table 1.

### ELISA

CXCL12 secretion by MM and BMSCs was measured using an ELISA kit (R&D Systems) according to the manufacturer's instructions. IFNγ production by polyclonally activated T cells was measured using the ELISA kit (eBioscience).

Macrophages were cultured in the absence or presence of MM cells (RPMI8226 and ARH77) for 48 hours, and then either stimulated or not with LPS (100ng/ml) (Sigma Aldrich) for an additional 24 hours. Cytokine production in macrophage and tumor cell supernatants was measured by the commercially available ELISA kits (TNFα and IL-10) according to the manufacturer's instructions (R&D Systems).

### Survival assay

RPMI8226 and ARH77 cells were stained with 5-(and 6)-Carboxyfluoresceindiacetatesuccinimidyl ester (CFSE) (5 μM, eBioscience) and cultured in the presence or absence of macrophages, in serum-full (10%) or serum-reduced (1%) medium and collected after 24, 48 or 72 hours incubation. Cell number was enumerated by FACS. Events were acquired during 30 seconds. Dead cells were eliminated by staining with PI. The relative number of viable cells in each sample was determined. To confirm the normalized flow rate and ensure accurate cell count, fixed cell concentration was counted prior to the experiment. BM samples (n=3) from MM patients containing CD138+ cells were cultured in 10% FCS medium in the absence or presence of macrophages for five days and percent of viable CD138+ PI-negative plasma cells was detected.

### Cell Cycle Analysis

MM cells that were incubated in the absence or presence of macrophages in serum-reduced (1%) medium for 48 hours were collected, washed with cold PBS, and fixed with 4% of paraformaldehyde (PFA) for 30 min. Fixed cells were resuspended in staining buffer containing 0.1% saponin (Sigma-Aldrich) and 40 μg/ml RNase and incubated at 37^0^C for 15 min. Cells were then stained with 10 μg/ml 7-amino-actinomycin D (7-AAD) (eBioscience) in dark for 30 min. DNA content was detected using FACS.

### XTT viability assay

ARH77 and RPMI8226 cells (5×10^4^ per 100 μl per well) were platedin 96-well flat plates in triplicates, with a different concentration of melphalan (5 μM) (Sigma Aldrich), bortezomib (2.5 nM) (LC laboratories) or lenalidomide (10 μM), in the absence or presence of macrophages for 48 hours. Cell viability was assessed using the 2,3-bis(2-methoxy-4-nitro-5-sulfophenly)-5-[(phenylamino) carbonyl]-2H-tetrazolium hydroxide (XTT) assay (Biological Industries).

### T cell activation and proliferation

T cell proliferation was determined using the CFSE-based assay. Macrophages were pre-cultured in the absence or presence of RPMI8226 cells for 48 hours, and excess of myeloma cells was removed by pipetting. Autologus lymphocyte-enriched PBMCs were thawed, labeled with CFSE (5 μM, eBioscience), plated in the absence or presence of macrophages and stimulated with anti-CD3 (OKT3) (10 μg/ml) and anti-CD28 (1 μg/ml) antibodies (eBioscience) for five days. Cell division was monitored by flow cytometric recording of the decrease in fluorescence intensity of CFSE-labeled cells. To calculate the proliferation index, the number of proliferated cells was divided by the number of the non-proliferated progeny.

### Statistical analyses

Data are expressed as the mean ± standard deviation (SD), or standard error (SE). Statistical comparisons of means were performed by a two-tailed unpaired Student's *t* test or the Mann-Whitney U test.

## SUPPLEMENTARY MATERIAL TABLE


