# Supplementary material for: Multiple myeloma cells recruit tumor-supportive macrophages through the CXCR4/CXCL12 axis and promote their polarization toward the M2 phenotype
Source: Oncotarget. 2014 Jul 12;5(22):11283–96. doi: 10.18632/oncotarget.2207 (PMC4294328; doi:10.18632/oncotarget.2207)
Supplement: Supplementary file 1 [file oncotarget-05-11283-s001.docx]

Multiple myeloma cells recruit tumor-supportive macrophages through the CXCR4/CXCL12 axis and promote their polarization toward the M2 phenotype

**Supplementary Table 1:**

| Gene |  | Primer sequence | product size |
| --- | --- | --- | --- |
| β2-microglobulin | sense | AGGCTATCCAGCGTACTCCA | 112 |
|  | antisense | TCAATGTCGGATGGATGAAA |  |
|  |  |  |  |
| CCL2 | sense | CCCCAGTCACCTGCTGTTAT | 135 |
|  | antisense | AGATCTCCTTGGCCACAATG |  |
|  |  |  |  |
| CCL5 | sense | GCTGTCATCCTCATTGCTACTG | 136 |
|  | antisense | TGGTGTAGAAATACTCCTTGATGTG |  |
|  |  |  |  |
| IL-8 | sense | CTGCGCCAACACAGAAATTA | 95 |
|  | antisense | ACTTCTCCACAACCCTCTGC |  |
|  |  |  |  |
| IL-1β | sense | CTGGTACATCAGCACCTCTCA | 154 |
|  | antisense | AGGGATTGAGTCCACATTCAG |  |
